# Intra- and interobserver agreement on the Oestern and Tscherne classification of soft tissue injury in periarticular lower-limb closed fractures

**Published:** 2014-12-30

**Authors:** Carlos Oliver Valderrama-Molina, Mauricio Estrada-Castrillón, Jorge Andrés Hincapie, Luz Helena Lugo-Agudelo

**Affiliations:** 1 Hospital Pablo Tobón Uribe, Medellín, Colombia; 2 Universidad de Antioquia, Medicine School, Rehabilitation in Health Group, Medellín, Colombia; 3 Universidad de Antioquia, Medicine School, Clinical Epidemiology Academic Group, Medellín, Colombia

**Keywords:** Soft tissue injuries, classification, tibial fractures, Reliability

## Abstract

**Background:**

The soft tissues injury in periarticular fractures of the lower extremities determines the proper time to perform bone fixation.

**Objective:**

The aim of this study was to determine the intra and interobserver agreement in the Tscherne classification.

**Methods:**

This is a descriptive, prospective study for patients admitted to the Pablo Tobón Uribe Hospital (PTUH) with tibial plateau or tibial pilon fractures. We performed a standardize evaluation using video photography at the time of admission and 24, 48, and 72 h after admission. Fifteen five reviewers who had various levels of training produced a total of 1,200 observations. The intra- and interobserver agreement was assessed using a weighted kappa for multiple raters and more than two categories.

**Results:**

Twenty patients were admitted with tibial plateau and tibial pilon fractures. The intraobserver agreement for all 15 raters was kappa 0.81 (95% CI 0.79-0.83), and the interobserver agreement for all 15 raters was kappa 0.65 (95% CI 0.55-0.73). The interobserver agreement at 24 h was kappa 0.67 (95% CI 0.46-0.86).

**Conclusions:**

Classifying the severity of soft tissue injury is critical in planning the surgical management of fractures of the lower extremities. Based on our results, we can reasonably argue that the Tscherne classification produced an adequate level of agreement and could be used to standardize and to guide the treatment, and to conduct research studies.

## Abstract (es)

**Antecedentes:**

La lesión de los tejidos blandos en fracturas periarticulares del miembro inferior determina el momento adecuado para realizar la fijación ósea.

**Objetivo:**

El propósito de este estudio fue determinar la concordancia intra e interobservador para la clasificación de Tscherne.

**Métodos:**

Estudio descriptivo, prospectivo en pacientes ingresados al Hospital Pablo Tobón Uribe con fracturas de platillos tibiales o pilón tibial. Se realizó una evaluación estandarizada utilizando videos y fotografías al momento del ingreso y 24, 48 y 72 h después. Quince evaluadores que tenían diferentes niveles de entrenamiento produjeron 1200 observaciones. La concordancia intre e interobservador fue evaluada utilizando kappa ponderado para múltiples evaluadores y más de dos categorías.

**Resultados:**

Veinte pacientes fueron admitidos con fracturas de los platillos o del pilón tibial. La concordancia intraobservador para los 15 evaluadores fue kappa 0.81 (95% IC 0.79-0.83), y la concordancia interobservador para los 15 evaluadores fue kappa 0.65 (95% IC 0.55-0.73). La concordancia interobservador a las 24 h del ingreso fue kappa 0.67 (95% IC 0.46-0.86).

**Conclusión:**

Clasificar la gravedad de la lesión de los tejidos blandos es un paso fundamental en la planeación del manejo quirúrgico de las fracturas periarticulares del miembro inferior, con base en nuestros resultados podemos afirmar razonablemente que la clasificación de Oestern y Tscherne tiene un nivel de concordancia adecuado y podría utilizarse de forma sistemática para estandarizar la evaluación entre los ortopedistas para orientar el tratamiento y también con fines investigativos.

## Introduction

The severity of the soft tissue injury plays an important role in the treatment of bone lesions, especially those that occur in areas with poor integumentary coverage, such as the proximal, diaphyseal, and distal portions of the tibia 1 ^,^ 2. Staged treatment has been favored for soft tissue injuries adjacent to tibial plateau and tibial pilon fractures. In the first stage of treatment, a temporary external fixation procedure allows the inflammation secondary to trauma to disappear; and in the second stage, final fixation procedures are completed with open or minimally invasive techniques 3 ^,^ 4. The results that have been published about staged treatment showed a decrease in complications associated with soft tissues (e.g., infection, wound dehiscence, and the need for flaps for coverage). With regard to tibial plateau fractures, these complications decreased by 80% in the eighties to approximately 5% at the beginning of the last decade 4. For tibial pilon fractures, complications decreased by 55% to 3.4% in the late nineties 5.

**Figure 1.**
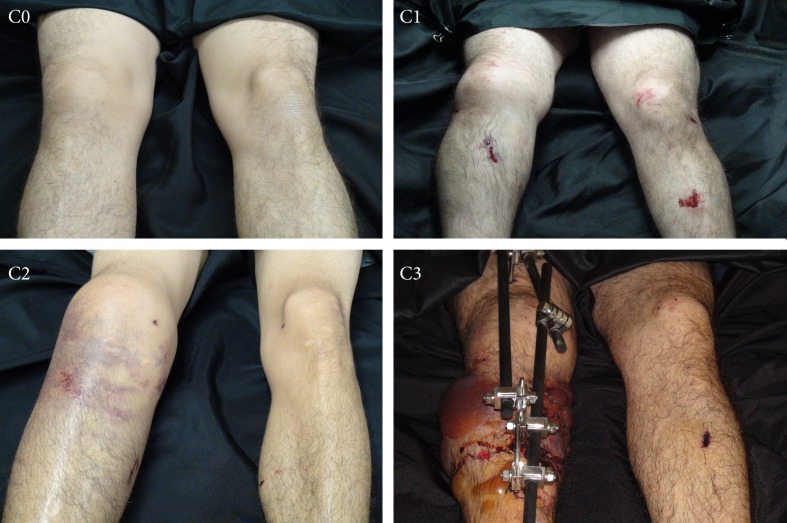
Oestern and Tscherne Classification. C0: Little or no injury to the soft tissues with simple fractures caused by indirect trauma. C1: Surface abrasions or contusions of the skin associated with simple or medium complexity fracture lines; these injuries are usually caused by the pressure exerted by the bone fragments on the soft tissue. C2: Deep contaminated abrasions and contusions in the skin and muscle resulting from direct trauma. Imminent compartment syndrome fits into this group. The Associated fracture lines are generally complex. C3: Extensive skin contusion, destruction of muscle or subcutaneous tissue and closed avulsions. Compartment syndrome and vascular lesions are included. The fracture lines are complex.

Therefore, determining the severity of the initial soft tissue injury is currently one of the most important factors in selecting the initial treatment: it helps the surgeon to make decisions about which bone fixation techniques must be used 6. However, the assessment of the severity of these lesions is usually subjective and differs between surgeons; for this reason, various classifications have been proposed to try to standardize the measurement of the extent of the integumentary injury 7. The classification used most often in the literature to describe the soft tissue injury in blunt trauma is the Oestern and Tscherne classification 8 ^-^ 10, which was published in the early eighties. This classification system rates the severity of blunt soft tissue injury on a scale from 0-3 (0 indicating little or no injury; 3 indicating the most serious injuries, including compartment syndrome and vascular lesions) (Fig 1). 

The relationship between the severity of injuries according to this system and long-term outcomes has been assessed ^1^; however, we are not aware of any studies that have evaluated intra- or interobserver agreement on the classifications. This gap in the literature has been considered one of the reasons why this classification system has not been applied more commonly in clinical practice 11. Another important aspect is that the associated soft tissue injury is progressive, and the inflammatory phase of trauma usually begins to abate 72 h after the initial trauma 6. Therefore, the level of agreement on the classification may be influenced by the point in time when the injury is classified. The ideal time point for using the Oestern and Tscherne classification to categorize the extent of soft tissue injury has not been determined.

The purpose of this study was to determine the intra- and interobserver agreement on the Oestern and Tscherne classification of soft tissue injury associated with closed periarticular fractures of the lower extremities (tibial plateau and tibial pilon), to describe how the agreement varies according to the level of experience of the reviewers and the time of the assessment.

## Materials and Methods

### Design

This is an observational, descriptive, prospective study to evaluate the intra- and interobserver agreement on the classification of consecutive patients who were admitted to the emergency department of the Pablo Tobón Uribe Hospital (PTUH) between January 2011 and April 2012. For the publication of this research, we adhere to the recommendation of the Guidelines for Reporting Reliability and Agreement Studies (GRRAS) 12.

### Patients

The patients included in the study were individuals between 16 and 60 years of age who lived in the metropolitan area of Medellín (Colombia). The patients had been involved in traffic accidents, had been diagnosed with closed tibial plateau or tibial pilon fractures (AO classification 41A2 to 41C3 and 43A1 to 43C3), and had been admitted to PTUH in the first 6 h after the initial trauma. The patients who did not meet the inclusion criteria or did not wish to participate in research were excluded from the study.

### Clinical and video photographic evaluation

At the time of admission to the emergency department, a signed informed consent form was obtained from each patient. The patients completed an admission card including the following variables (among others): age, sex, mechanism of trauma, history of hypertension and diabetes, and smoking status. Limb circumference was measured at the level of the tibial tuberosity for the tibial plateau and 3 cm from the tip of the medial malleolus for the tibial pilon (both compared with the contralateral limb). Additional measurements were taken of abrasions, blisters, and, bruises and their locations on the injured limb were recorded. These measurements were repeated after 24, 48, and 72 h. Video photographic images of the patient's limb were obtained at the time of admission and after 24, 48, and 72 h. A Sony high definition (1080i) HDR-HR520 camera was used to obtain the images. For the images, the injured limb was positioned on a standard black background with no markings. Only images of the limb were obtained; no other elements were included that would enable the identification of the patient. The camera was placed on a tripod that allowed a perpendicular view of the limb at a distance of 50 cm. Videos of the anterior sides of both limbs and the medial and lateral sides of the injured limb were recorded, and 12-megapixel photographs were taken at a minimum distance of 50 cm from the anterior, medial, and lateral sides of the injured limb. The person responsible for taking these images was a general practitioner who received basic training from an expert in photography to standardize the amount of light and master the macro and other technical features essential for taking the images.

The same procedure was repeated in the inpatient unit of PTUH 24, 48, and 72 h after trauma to observe the progress of the soft tissue injury.

The videos and photos were edited to create one minute video photographic packages, resulting in 4 different assessments for each patient. These packages were stored on a laptop that was exclusively used for this research, and a back-up copy was made on a 1-terabyte capacity external hard drive. 

Information about this research and the importance of the Oestern and Tscherne classification was disseminated to the orthopedists and residents at the institution using posters beginning one month prior to the enrollment of the patients.

### Evaluators

For the assessment of intra- and interobserver agreement, five third- and fourth-year residents in the orthopedics program at Bolivariana Pontifical University were invited to participate. In addition, five orthopedists from PTUH with experience in the treatment of tibial plateau and tibial pilon injuries were invited to participate. These orthopedists had treated five to 10 cases of these fractures in the last year, or they had treated these injuries in the emergency room. They were not the treating physicians of the patients in this study and had not had direct contact with these patients. Five orthopedists that mainly worked outside trauma and conducted more than 20 surgical procedures for these types of fractures each year were also invited to participate. Thus, a diverse group of evaluators was recruited to apply the Oestern and Tscherne classification system: five inexperienced practitioners (residents), five typical users (orthopedists), and five experts (orthopedic trauma surgeons). The number of evaluators at each level of experience was determined based on the empirical recommendation by Audige *et al* 13. 

### Evaluation sessions

Each evaluation session included four patients (i.e., 16 video photographic packages) and lasted for 16 minutes to avoid fatigue in the evaluators. When all of the video photographic assessments for a group of four patients had been completed, a PowerPoint^®^ 2007 presentation was prepared with the images for a session. Each package was organized randomly so that no evaluator reviewed the same sequence of cases. Each session took place in an academic area lounge of the hospital with one evaluator. There were no other people in the room except the person who organized the image display. This procedure guaranteed the independence of the evaluations. The images were shown on a Sony^®^ HD 42" TV; the evaluator was positioned in front of the television at a maximum distance of 1.5 m, and the laptop was connected to the TV with an HDMI cable. No clinical information was given to the evaluator about the cases. The evaluator had a maximum of one min to record their results on the card designed for this purpose. To assess intraobserver agreement, the session was repeated after 10 to 14 days under the same conditions as described above with the same array of cases; however, a new random order of the packages was used to avoid recall bias. 

### Bias control

The subjects of the present study were patients who were admitted to the emergency department of PTUH with periarticular lower-limb fractures; bias at this level was controlled with the inclusion and exclusion criteria. Standardizing the video-photography technique controlled bias related to the object; however, this type of assessment may differ from a clinical evaluation in the emergency setting. As a result, the initial evaluation performed in the emergency room by the shift orthopedist who admitted the patient was used as an additional variable to compare with the video photographic assessments. To prevent the evaluators from being influenced by the responses of another evaluator, the sessions were designed as individual sessions. To avoid recall bias in the assessment of intraobserver agreement, an interval of 10-14 days between assessments was used. For the same purpose, the video matrix was randomly assigned for each session and each evaluator. 

### Statistical analysis

In each evaluation session, the results were recorded in a document designed for that purpose. For quantitative variables, descriptive statistics were used (i.e., means with standard deviations and medians with interquartile ranges). For qualitative variables, ratios were used. For the analysis of intraobserver agreement, the kappa statistic was used (weighted by quadratic weights). For the analysis of interobserver agreement, the kappa statistic was used (weighted by quadratic weights) with more than two evaluators and more than two categories using the method proposed by Fleiss 14. The kappa values with corresponding confidence intervals are shown by classification category, by time point (admission, 24, 48, and 72 h), and by level of training. The kappa index values were classified using the system proposed by Landis and Koch 15: 0=poor; 0.01-0.2= mild, 0.21-0.40= fair; 0.41-0.60= moderate; 0.61-0.80= substantial; 0.81-1.0= almost perfect.

To calculate the sample size, the formula revised by Ciccetti 16 was used to apply the weighted kappa index to an ordinal scale with four categories (as the Oestern and Tscherne classification system uses). In this formula (2k^2)^, K represents the number of categories on a nominal scale. For the Oestern and Tscherne classification system (with four categories), the formula 2(4^2)^ implies that 32 cases are required for the planned video photographic evaluation. 

The software used for the descriptive statistics was SPSS^®^ v17. To calculate the intra- and interobserver agreement (kappa) and the 95% confidence intervals, AGREESTAT^®^ 2011 was used 17. To calculate the grouped intraobserver kappa and the respective 95% confidence intervals, EPIDAT^®^ V4 was used 18.

The study had the approval of the research and ethics committee of PTUH and the Medical Research Institute of the University of Antioquia, and each patient signed an informed consent form to undergo the imaging studies.

## Results

Between February 2011 and February 2012, 67 patients were admitted to PTUH with tibial plateau and pilon fractures. Of these patients, 40 did not meet the inclusion criteria (21 patients were admitted more than six hours after trauma, 17 patients had non-displaced fractures and were discharged immediately, one patient was deaf and had no companion to consent at study entry, and one patient was in critical condition with multiple trauma). Seven patients who met the inclusion criteria did not enter the study because the authors were not alerted to take the images (five patients) or because no one was available to record the videos (admission in the early morning hours; two patients). Twenty patients entered the study (14 men; mean age 33.9 years). Eighteen patients had tibial plateau fractures, and according to the AO classification, eight cases were 41C, and 10 cases were 41B3. The two cases of tibial pilon fractures were classified as AO 43C1. Sixteen fractures were immobilized with noninvasive methods (plaster splint: three; brace: 13) and four cases received external fixation. All of the patients required open reduction and internal fixation for the treatment of their bone lesions, and there were no complications related to the soft tissues in any of the cases during the postoperative follow-up period (mean follow-up: 11.7 months (SD 3.4)).

Eighty video photographic packages were obtained (4 for each patient). During 150 testing sessions (75 inter- and 75 intraobserver), 1,200 classification measurements were recorded, 300 measurements for each time point (admission, 24, 48, and 72 h). Each group of evaluators (inexperienced practitioners, regular users, experts) conducted 50 evaluation sessions (25 intraobserver and 25 interobserver sessions), for a total of 400 assessments per group. The agreement between the in-person admission evaluation in the emergency room and the video photographic assessments was 0.63 (95% CI 0.55-0.70).

The overall intraobserver agreement for all 15 evaluators was 0.81 (95% CI 0.79-0.83), which corresponds to "almost perfect" intraobserver agreement according to the system proposed by Landis and Koch for kappa classification (Table 1). 

**Table 1.**
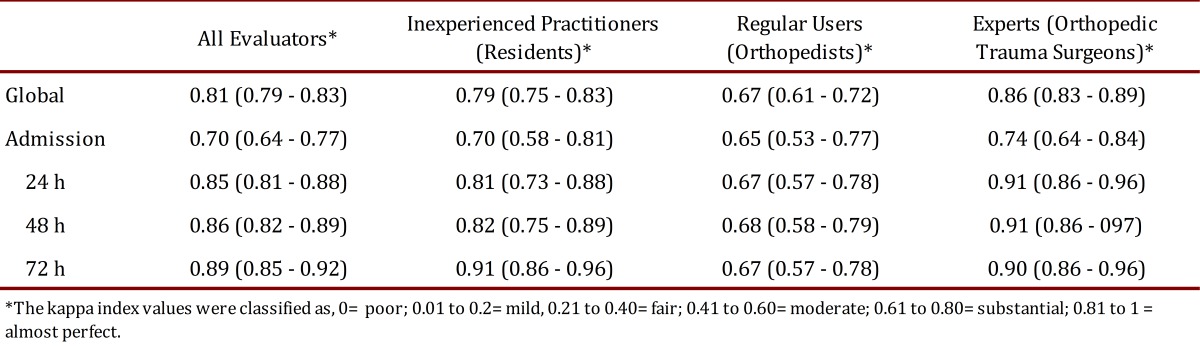
Intraobserver agreement (Kappa (95% CI)).

The overall interobserver agreement for all 15 evaluators was 0.65 (95% CI 0.55-0.73), which corresponds to "substantial" interobserver agreement according to the system proposed by Landis and Koch for kappa classification. The interobserver agreement for each group of evaluators and each evaluation time point can be seen in Table 2. 

**Table 2.**
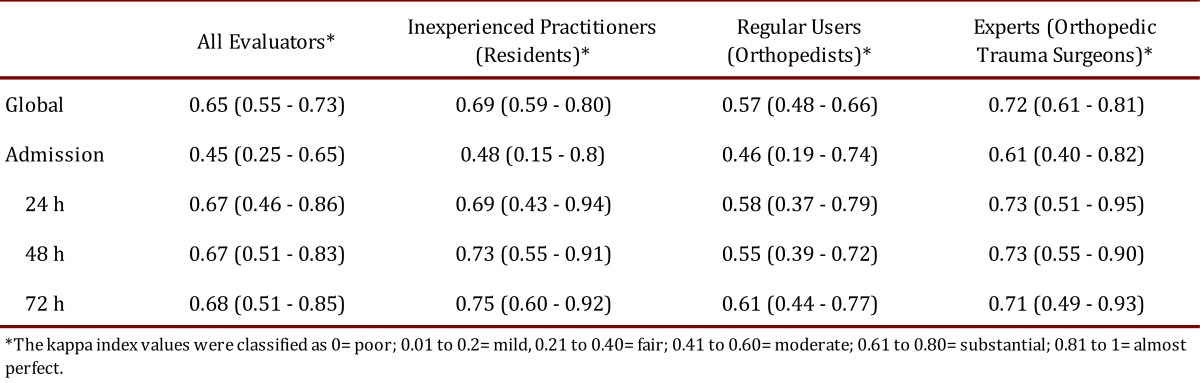
Interobserver agreement (Kappa (95% CI)).

## Discussion

The severity of the soft tissue injury in patients with periarticular fractures of the lower extremities is the main factor for determining the proper time for surgical treatment. Several classification systems have been described to assess the severity of soft tissue injury, and the Oestern and Tscherne classification is the simplest and most commonly used system in the orthopedic literature. Its association with postsurgical complications has been described 19 ^,^ 20. However, we do not know of any previous studies that have evaluated intra- and interobserver agreement for this classification system. In this study, we found "almost perfect" intraobserver agreement and "substantial" interobserver agreement using the Landis and Koch system for classifying agreement when using the kappa statistic. 

The intraobserver agreement was excellent in this study. The group of orthopedists had the lowest level of agreement (kappa 0.67, 95% CI 0.61-0.72), but even for this group, the agreement was "substantial". This group included an upper limb surgeon, an orthopedic oncologist surgeon, and three orthopedists with less than five years of experience. Intraobserver agreement was the highest in the orthopaedic trauma group (kappa 0.86, 95% CI 0.83-0.89); this group consisted of two knee surgeons with over 10 years of experience, one foot and ankle surgeon with over five years of experience, and two trauma surgeons with over five years of experience. The group of residents showed intraobserver agreement beyond what was expected: their agreement was higher than the orthopedists and very close to that of the orthopaedic trauma surgeons. The differences between the levels of agreement can be explained by the level of familiarity with the classification and the need to use it. For the group of orthopaedic trauma surgeons, it is essential to determine the severity of soft tissue injury because it defines the type of treatment. For the group of orthopedist, the classification system may not be as relevant, and their interest in using this system or any other classification system may be low. The group of residents has been exposed to the classification system throughout their training and has been specifically trained in its use, which could explain the results obtained in this group. 

The intra- and interobserver agreement at the four evaluation time points shows that the agreement at the time of admission (<6 h after trauma) was almost always lower than at the other three time points. In the evaluations after admission, intraobserver agreement remained at the "almost perfect" level defined by Landis and Koch, and interobserver agreement remained at the 'substantial' level. Because soft tissue injury is progressive, it would seem that determining the severity of the injury at the time of admission is not easy. Only the onset of more definite signs 24 h after trauma (e.g., bruising, blisters, increased edema, or other signs) would enable more accurate and consistent classification. 

This is the first study to evaluate the agreement on the Oestern and Tscherne classification, and it obtained satisfactory results, unlike other studies that have evaluated agreement on other classification systems. A study on the Gustilo and Anderson classification of open fractures reported an agreement percentage of only 60% 21, and agreement studies on fracture classification systems in radiology found a kappa value of 0.72 for the Young-Burgess classification system and a kappa value of 0.40 for the Tile classification 22. Most studies that evaluate interobserver agreement have common methodological 13 problems. To solve this problem, the GRASS guide was recently developed ^12^ to standardize the published results of agreement studies, and we followed this guide. We believe that we were able to avoid most of the methodological difficulties described in the guideline proposed by Köttner *et al* 12. The evaluations were independent because they were performed individually. We address the recall bias by organizing the presentations in random sequences, which ensured that an evaluator would never see the same images in the same order. Furthermore, we left a minimum of 10 days between the intraobserver assessments. We used the kappa statistic (weighted by quadratic weights), calculated using the Fleiss method for ordinal classification systems, such as the Oestern and Tscherne system.

The use of videos and photographs as valid tools for evaluating agreement has been widely described in dermatological literature, with "almost perfect" agreement between live assessments and video photography assessments 23 ^,^ 24. However, there are few studies in the orthopedic literature that use video photography to assess the agreement between raters in a specific classification. One of these studies assessed the percentage of agreement in the Gustilo classification of open fractures 21. In our study, the agreement between the live assessments and the video photography assessments was 0.63, similar to the interobserver agreement found for all 15 evaluators. 

Some limitations have to be acknowledged. First, the sample size was calculated to determine the number of cases necessary; however, we did not reach 32 cases, which impacted the accuracy of the results, especially the evaluations at each time point. However, by grouping the 80 evaluations as a "global" item, there were enough cases, and we managed to obtain accurate and informative results. Second, the difference between the clinical and the video assessment of the patient may lead to an information bias. However, the design features of our study guaranteed that all the raters were in the same condition when making the video photography assessments. In addition, given the limitations to conduct a study like this in a real clinical setting, it seems that the strategy we used is the best option to assess the agreement of this type of classifications. Finally, like any agreement study, we cannot generalize these results because they are influenced by cultural, social, and educational factors that may not be replicated in other settings.

## Conclusion

Classifying the severity of soft tissue injury is a critical step in planning the surgical management of periarticular lower-limb fractures. Based on our results, we can reasonably state that the Oestern and Tscherne classification system produced a satisfactory level of agreement and can be used for standardizing the assessments of orthopedists who routinely treat these injuries and for research purposes. The association between the severity of soft tissue injury according to the Oestern and Tscherne classification and postoperative complications should be tested in a prospective multicenter study with an adequate sample size that controls for other factors associated with these complications. 

## References

[1] Dirschl  DCL (2010). Classification of fractures.

[2] Volgas  D (2011). Preoperative assessment and classification of soft-tissue injuries.

[3] Sirkin M, Sanders R, DiPasquale T, Herscovici DJr (2004). A staged protocol for soft tissue management in the treatment of complex pilon fractures. J Orthop Trauma.

[4] Egol KA, Tejwani NC, Capla EL, Wolinsky PL, Koval KJ (2005). Staged management of high-energy proximal tibia fractures (OTA types 41): the results of a prospective, standardized protocol. J Orthop Trauma.

[5] Thordarson DB (2000). Complications after treatment of tibial pilon fractures: prevention and management strategies. J Am Acad Orthop Surg.

[6] Tull F, Borrelli JJr (2003). Soft-tissue injury associated with closed fractures: evaluation and management. J Am Acad Orthop Surg.

[7] Südkamp  NP (2007). Soft-tissue injury:.

[8] Oestern HJ, Tscherne H (1983). Physiopathology and classification of soft tissue lesion. Hefte Unfallheilkd.

[9] Oestern HJ, Tscherne H (1983). Pathophysiology and classification of soft tissue damage in fractures. Orthopade.

[10] Tscherne H, Oestern HJ (1982). A new classification of soft-tissue damage in open and closed fractures (author's transl). Unfallheilkunde.

[11] Sirkin  SM, Liporace  M (2009). Chapter 14: Fractures with soft tissue injuries.

[12] Kottner J, Audige L, Brorson S, Donner A, Gajewski BJ, Hrobjartsson A (2011). Guidelines for reporting reliability and agreement studies (GRRAS) were proposed. J Clin Epidemiol.

[13] Audige L, Bhandari M, Kellam J (2004). How reliable are reliability studies of fracture classifications? A systematic review of their methodologies. Acta Orthop Scand.

[14] Fleiss JL, Levin BA, Paik MC (2003). Statistical methods for rates and proportions.

[15] Landis JR, Koch GG (1977). The measurement of observer agreement for categorical data. Biometrics.

[16] Cicchetti DV (1981). Testing the normal approximation and minimal sample size requirements of weighted Kappa when the number of categories is large. Appl Psychol Measur.

[17] Gwet  KL AgreeStat 2011.

[18] Epidat V4.0 2012.

[19] Hoiness P, Engebretsen L, Stromsoe K (2003). Soft tissue problems in ankle fractures treated surgically. A prospective study of 154 consecutive closed ankle fractures. Injury.

[20] Gaston P, Will E, Elton RA, McQueen MM, Court-Brown CM (1999). Fractures of the tibia. Can their outcome be predicted?. J Bone Joint Surg Br.

[21] Brumback RJ, Jones AL (1994). Interobserver agreement in the classification of open fractures of the tibia. The results of a survey of two hundred and forty-five orthopaedic surgeons. J Bone Joint Surg Am.

[22] Koo H, Leveridge M, Thompson C, Zdero R, Bhandari M, Kreder HJ (2008). Interobserver reliability of the young-burgess and tile classification systems for fractures of the pelvic ring. J Orthop Trauma.

[23] Rimner T, Blozik E, Fischer Casagrande B, Von Overbeck J (2010). Digital skin images submitted by patients: an evaluation of feasibility in store-and-forward teledermatology. Eur J Dermatol.

[24] Ribas J, Cunha Mda G, Schettini AP, Ribas CB (2010). Agreement between dermatological diagnoses made by live examination compared to analysis of digital images. An Bras Dermatol.

